# Medical and Philosophical Intelligence

**Published:** 1817-06

**Authors:** 


### MEDICAL AND PHILOSOPHICAL INTELLIGENCE.

THE last Number of the Repository contains a paper from Mr. Cross, exactly confirming our opinion concerning the attempted improvement of silk ligatures cut close to the knot. After amputation of the thigh in the human subject, and several experiments on a dog, the writer concludes—

"The experiments which I have detailed, have, in some respects, afforded different results from those which Mr. Lawrence made, and lead to the following conclusions, which are not very favorable to the employment of the minute silk ligatures after operations.

"1st. If the wounds do not unite by the first intention, the ligatures may escape with the discharge, without any inconvenience.

"2d. If common ligatures of twine are cut short, the wound may unite over them, and they may be found in abscesses after an interval of many weeks.

"3d. If the finest dentist's silk be employed in the same way, the wound uniting over it, the ligature may be detached from the vessel, and remain buried in an abscess, where it will be found at different periods, from one to seven months; and this may happen, whether the vessel be firmly compressed with a single ligature, or divided between two ligatures, so as to imitate the circumstances under which vessels are tied after operations.

"5th. If Indian silk, fine as a hair, be put round a vessel, so as diminish its diameter, or to effect its obliteration, by just compressing its sides together, it may remain in this situation without exciting abscess, or producing any inconvenience. The ligature may be thus applied to compress an artery for the cure of aneurism; but not to secure vessels divided by operations. If a thin ligature be drawn sufficiently tight upon a vessel on the face of a stump to be secure, I am persuaded that the extremity of the vessel, which becomes insulated, as it were, must die. How often do we see this slough of the main artery of the thigh come away with the ligature!"

"Ought the surgeon readily to give up the comfort of knowing that all the ligatures, particularly the one from the main artery, are away? Have the minute short ligatures any other advantages than that of avoiding the irritation of threads hanging out of the wound? Are there not sufficient objections to their being employed generally in practice, however applicable to particular cases? May not the surgeon, who is less frequently engaged in operating, include with the artery a neighbouring nerve?

"I conclude with these queries, under the hope that those surgeons, who have been captivated with Mr. Lawrence's ingenious proposal, may answer them by giving publicity to their experience upon the subject."

---

The *Royal Society* has lately been particularly barren in natural history. One of the papers has, however, occupied, if we may so say, "all the talents." Sir Joseph Banks procured some fossil bones from the Catwater at Plymouth. Sir Everard Home and Mr. Brooks determined that some of them belonged to a rhinoceros larger than the one in Mr. B.'s possession, which is the largest in England. Lastly, Mr. Brand found them to consist of phosphate and carbonate of lime, besides animal matter $\frac{2}{100}$, and water $\frac{10}{100}$.

The Rev. Mr. Hyde Wollaston's thermometer for measuring the heights of mountains by the boiling point of water, promises some useful analogical discoveries.

Mr. Marshall's paper on the laurus cinnamomun is chiefly interesting on account of the etymology of the word. The Malays, we are informed, express cinnamon by the word *cayeu menes*, sweet wood, which, as it is well known the Romans pronounced the *c* sharp, and gave their own termination to every foreign word, was easily transferred to *kin* or *cin-cinnamomum*. Cassia, Mr. Marshall conceives, is derived from the same origin.

---

The *London Medical Society* closed their meetings for the season, on Monday, the 26th of May. A paper was read from Mr. DUNN, describing his successful operation in a scrofulous ankle. He dissected out some of the tarsal and metatarsal bones which were diseased, preserving the toes and the rest of the foot. In a few months, the subject being in the vigor of youth, sufficient new matter was formed to restore the figure and usefulness of the foot.

On

On the same evening, a paper of Dr. CLUTTERBUCK's was read, remarking that fevers, from which the metropolis had been free for many years, were becoming sporadic, and even were increasing in frequency; that they were scarcely reducible to any particular character, (such as the French call *ataxique*,) and had, in some cases, proved fatal at very uncertain periods: as they did not seem infectious, that they might probably be imputed to the same unknown cause in the atmosphere as has proved so unfavorable to vegetation.

---

*Medical Benevolent Society.*—At the first annual general court, the following gentlemen, Dr. Luke, Dr. Drever, Joseph Hayes, Esq., A. T. Thompson, Esq., R. S. Wells, Esq., and Reginald Williams, Esq., went out of the direction by rotation; and Dr. G. G. Currey, Dr. Outram, H. L. Thomas, Esq., R. R. Pennington, Esq., Neville Wells, Esq., and Edward Browne, Esq., were elected.

The number and names of members, and the accounts of the society were reported, and the 10th of May appointed for the anniversary festival.

The first anniversary of this admirable institution was accordingly commemorated at the Freemason's Tavern, on Saturday, May the 10th, when, between forty and fifty gentlemen, among whom were some of the most distinguished members of the profession, assembled: The President of the Royal College of Physicians, Dr. LATHAM, to whose philanthropy the society owes its foundation, in the chair.

After the cloth was drawn, the worthy chairman took occasion, among other observations equally creditable to his feeling and good sense, to express the peculiar gratification he derived from the attendance of so large a portion of the members of this infant society; which he interpreted as a proof of their zeal in promoting its welfare, and as an auspicious omen of its future prosperity.

Although the present number of its members bore a very small proportion to that of the profession, yet when he remembered the degree of support which similar societies had met with on being first instituted, and when he saw the amount of the contributions already bestowed, the *Medical Benevolent Society* might be considered in a prosperous condition. The state of the funds indeed was highly satisfactory. The expenditure would of course appear large, but it would be recollected that this was the year of the society's being established, when the expences were necessarily greater than any future year would require.

It would be seen, by reference to the plan of the Society, that two modes offered by which a comfortable provision might be secured in case of any of those vicissitudes in life to which medical men were peculiarly exposed, or when age and infirmity rendered a member unequal to further labour:—the one was by paying annually one guinea or more, till he have paid the sum of thirty

 guineas, guineas, which would entitle him to such relief as, upon application, the funds would afford; the other was by paying at one time a sum of money, which at twenty-five years of age was £49 only, or annually a small premium, which would, at the age of sixty, secure the absolute property of an annuity of at least £50 for the remainder of life.

When the existence and views of this society were more generally circulated, he was confident that it would have the approbation and support of the more affluent members of the profession.

Several liberal donations, both from professional and unprofessional friends to the institution, were received, and many new members were proposed. The impressions ever resulting from the consciousness of good actions, together with the liberal and unremitting attentions of the stewards, were so gratifying, that the day concluded with general satisfaction.

---

Some ingenious remarks on flame have lately attracted the notice of experimental philosophers; the result at present is, that in most, if not all, flames there is an exterior and more feeble flame, which is in general invisible, on account of the internal more brilliant flame. That the exterior flame is the part really undergoing combustion and giving out heat. [Quere.—What analogy has this to the calorific and colorific rays of the sun discovered by Herschell ?] That flame is not opaque, and only obscures every subject beyond it by its greater brightness. The following are among the proofs of the above, and contain Mr. Porrett's general conclusion.

" Place a lighted spirit-lamp about three feet from a sheet of white paper stuck against a wall, and about nine feet from a lighted candle, so that the lamp may be between the wall and the candle. Close by the side of the flame of the lamp put a small piece of thin and clear glass. Then look at the sheet of paper, and two very faint shadows will be seen upon it: the one will represent the flame of the spirit-lamp, and the other the piece of glass; but the shadow of the glass will be the deepest: consequently the flame intercepts less light; or, in other words, is more transparent than glass.

" The opacity of flame will not now, I presume, be urged against the explanation which I have given of one of the obscuring effects of the wick. I proceed now to mention the other, which depends on its conducting power. A long wick conducts downwards a considerable portion of the heat of the flame, which is expended in volatilizing more tallow (hence the increased consumption of that substance); and, by thus diminishing the temperature of the flame, the particles of charcoal are not ignited to such an intense degree as they otherwise would be, and consequently they do not emit so much light. Hence whatever conducts off much caloric from the flame diminishes its light. I have been able to reduce the temperature of the flame of a candle so considerably by placing it in an impure atmosphere, at the same time that a metallic sphere of the size size of a musket-ball was suspended so as to dip deep into the flame, that it burned with a feeble blue light, like that of spirits of wine. A similar effect takes place at the bottom of the flame of a candle: the small quantity of gas and vapour emitted from this part of the wick burns here with a feeble blue flame, in consequence of its proximity to the wick replete with tallow passing to the gaseous state, by which its temperature is kept down. In the lighted stream of coal-gas the same cooling effect is produced near the bottom by the constant current of cold gas. In all these cases the heat of these parts is not sufficient to decompose any portion of the gas inclosed within the outer flame; consequently no charcoal is separated and ignited; and therefore (conformably to the principle discovered by Sir H. Davy) the emission of light is very trifling."—*Annals of Philosophy.*

The same work contains the Chinese clumsy mode of preparing mercury in its various oxids and in ointments. They are only worth notice as marking the character of that extraordinary nation.

An ingenious paper, by Mr. Sym, on the Temperature at which Water is at the greatest Density, concludes thus,—

"The result of my trials, which I repeated oftener and more scrupulously than an older experimenter would have thought at all necessary, is conformable to the generally received opinion. Whether I plunged the bulb into water at 50°, and then slowly cooled it down to 32°, maintaining the temperature for some time stationary at each successive interval of two or three degrees, in order to make sure of its being uniformly diffused throughout both members of the instrument; or whether I reversed the process, by first surrounding the bulb with ice, and then slowly communicating heat to it; I found that the condensation was greatest either at 40°, or in its near neighbourhood, and that the rate of expansion on each side of that limit was apparently the same. I even made the experiment with a greater degree of nicety than can be done with a graduated scale, by tying a fine hair round the tube, and placing it precisely opposite to the level of the water, after having kept it at 40° for more than half an hour; and I still found that any change of temperature, whether a rise or a fall, caused the water to ascend above the level of the hair. I am, therefore, almost convinced that the greatest density of water is at a temperature not differing from 40° by more than one degree; though I should not like to speak confidently on the subject, unless I could have the satisfaction of seeing the method approved, and the result verified, by some more experienced chemist."

---

Professor Leslie has found that the dried garden mould and decayed green-stone pulverised, will "absorb the 50th part of its weight of moisture before its absorbing power is diminished one half, and the 25th part of its weight before this power is reduced to one-fourth.

one-fourth. When completely saturated with humidity, it may hold near a fifth part of its whole weight. Since, therefore, the quantity of heat abstracted by the process of evaporation is adequate to the congelation of about eight times an equal weight of water, the dry pulverized green-stone or garden mould is capable of freezing more than the sixth part of its weight of water. To ensure success and expedition, however, I should prefer rather a larger proportion of the powder. The contents of two quart decanters, for instance, poured into a saucer of a foot diameter, might be employed to freeze half or three quarters of a pound of water in a hemispherical cup of porous earthenware. This powder is capable of acting still, though with feebler effect. It should, therefore, after each process, be dried again, which will restore it completely to its former energy. Such partial drying will be quickly and easily performed; and in hot countries the mere exposure of the powder for a while to the sun may be sufficient. Ice may, therefore, be procured in the tropical climates, and even at sea, with very little trouble, and no sort of risk or inconvenience."

---

Some experiments have been made of inoculating cows with variolous matter, but no contagious marks have followed. Twelve years ago, we made a similar experiment with the same results.

---

*Extract of a Letter from Edinburgh.*—"Dr. Spurzheim sank not under this cruelty of criticism, which he bore with a serenity of deportment worthy a man of science. On the contrary, his moral character appeared more bright in the eyes of those who knew him, simply from being contrasted with the foulness of the epithets that had been thrown upon it. He came to Edinburgh, therefore, not to indulge feelings of personal irritation, but in a spirit of meekness, anxious to find out his opponent, for no other purpose, than that he might convince him, by ocular demonstration, of that peculiar structure of the brain, which he had described in his works and plates.

"I had the good fortune to be present at his first demonstration, which took place before a considerable number of eminent anatomists; the person also was there, whom rumour alleges to be the author of the offensive article in the Review. I marked the conduct of that individual, and, if the outward deportment could be viewed as an indication of what was passing in the mind, he was certainly labouring under suppressed emotion; and more than once he tried to disembarrass himself by pulling from his pocket, and reading, or pretending to read, the superscription of a letter. He generally contented himself with distant hurried glances at what was demonstrated, and, upon the whole, seemed both uneasy and inattentive."

"On a subsequent occasion, Dr. Spurzheim, with his usual readiness, diness, demonstrated the brain to about two hundred spectators, among whom were several of the medical professors, and other competent judges. It was previously concerted, that his opponent should ask him questions; and, by so doing, it was hoped, to give him and his doctrines a public and final overthrow. The scene was most interesting to the audience. Dr. Spurzheim, in his usual masterly manner, proceeded in the demonstration, and like the 'admirable Crighton,' sustained, for upwards of four hours and a half, and in a language which was foreign to him, a public disputation with his adversary, explaining himself in terms at once philosophical and perspicuous, and very successfully and coolly ridding himself of the disingenuous cavilling about words with which it was sought to embarrass him. During this public disputation, it is pretty generally admitted, that there was, in one quarter, a right plentiful lack of temper, as well as of argument, and that had Rare Ben Johnson been alive to witness the scene, he might have found hints for improving one of his best characters, viz. that of the 'Angry Boy' in the 'Alchymist.'

"I am thus particular in my statement, because attempts have been made to misrepresent the feelings and judgment of the audience, which were unquestionably in favor of Dr. Spurzheim, in the proportion of at least twenty to one. It would have been too much to expect perfect unanimity on a question and an occasion of this sort; for little does he know of human nature, who is not aware what erroneous impressions on 'the mind's eye' the *mirage* of prejudice and predilection now and then produces."—*Med. Chir. Journ.*

---

We have not thought it necessary to notice the late attempt at curing cutaneous diseases, (some of our readers will, perhaps, prefer the term pseudo-syphilitic), by nitro-muriatic baths. Our neighbours on the continent, it is well known, are fond of more complicated pharmacy.

Dr. Kretschmar, of Dessau, highly recommends the use of uligenous gas baths, which gas is the produce of putrified vegetable and animal matter. By the former, carbonic, hydrogen, and carbonic acid gas, are produced; and, by the latter, phosphoric hydrogen gas, to which, in turfy countries, comes still hepatic gas. Water impregnated with these substances must naturally be expected to be very active; there is, however, still another reason that must render these baths salutiferous, viz. the electricity evolving by these decompositions. They would in general be applicable in palsy, stiffness and wasting of the limbs, nervous debility, &c.

The construction of such a bath would require a grate in a place beneath the water, where this gas is evolved in the greatest quantity, and that it be covered with a board having holes bored in it.

---

*Remarkable case of total Obliteration of the Aorta.*—In the corpse of a youth fourteen years of age, that had laboured under violent violent palpitation of the heart and dyspnœa, whose lower extremities had not attained the same degree of perfection as the superior ones, a disproportion was discovered between the ascendant and descendant aorta; the former being found distended into a sack of nearly four inches in circumference, and a similar dilatation existing in the heart itself, and in all the ramifications of the ascendant aorta, so that the left subclavian in particular was found twice as large as usual, and appeared like a continued aorta; but the descending, much contracted and straightened in the beginning, and afterwards, about six or seven lines beneath the subclavian, entirely obliterated,—soon, however, appearing hollow again, spreading its ramifications, and then retaining its usual permeability. The circulatory communication had probably been kept up by the superior intercostal arteries of the left side of the subclavian and the arteria mammarum by their intercostal branches, as all of these appeared much dilated. The arterious duct was likewise hollow, and wide enough to admit a catheter. The substance obliterating the aorta, resembled the ligamentous cellulous matter into which the arterious duct is generally changed after birth.

---

*Remarkable Instance of Obesity.*—Dr. Digby, in a letter to Dr. Rehmann, gives the following description of two enormously fat people,—David Eustinphejef, of the village Wladimirooka, in the Makareswick district of the government Nischney Novogorod; and his sister, Anastasia. David, fourteen years of age, stands four feet and six inches, English measure; weighs, without his clothes, three hundred weight and ten pounds; the circumference of his head, above his eye-brows, is one foot nine inches; across the middle of the face, one foot ten inches and a half. His forehead is short, roundish, flat, and thickly covered with light hair. His little brown eyes are deeply buried beneath the prominent eye-brows, yet they have a friendly childish look. The nose is somewhat flattened towards the root, and is rather blunt. The cheek-bones are prominent, and their farthest extremities distant six inches from each other. His physiognomy is pleasing, attractive, and natural. His white teeth, fresh and fair complexion, increase his tender and childish appearance. His neck is hardly perceivable; the breadth of his shoulders perfectly agrees with the widest diameter of his head. The circumference of his trunk beneath his arms is four feet and a half, in the epigastric region four feet. His breasts hang down like two enormous cushions of fat. The thickness of his upper arm near the insertion of the deltoid muscle is one foot seven inches, in the middle of the fore-arm one foot and one inch; of the thickest part of the thigh two feet and ten inches; above the knee two feet; and round the calf of the leg, one foot and eight inches. The parts of generation, still without hair, are almost totally covered by fat, suspended from the pubic region.

 The

The testicles are extremely small and hardly to be felt; the penis is also proportionably small.

His sister, Anastasia, twelve years of age, only differs from him in respect of a few pounds weight and a few inches in size. She also appears to be a little livelier than her brother, who discovers no sense for any thing besides eating and drinking.

---

Our correspondent at Paris has transmitted us the analysis, inserted in one of the London Journals, of intestinal gas. We shall spare our readers the trouble of reading it, but present them with our friend's note.

Every credit is due to the accuracy and care of Messrs. Chevreuil and Majendie, and we have no doubt whatever as to the result of their important experiments being as described; but we would put one question to the learned author of this memoir, and that is connected with the history of the sciences. The gases, we are taught to believe, were very imperfectly known before the time of Priestley; how comes it then, that a person in 1489 appears not only to be acquainted with all the gases, but also with the nomenclature which the chemists of the eighteenth century claim the merit of inventing? That the science might have recognized combined products of the nature of the gases determined by the modern system of analysis is probable; but, in the then state of chemistry, we hesitate not to affirm, that analysis was so very imperfectly known that it was impossible to arrive at any thing but confused notions on the subject; as, for instance, what would have been the value of the experiments even of M. Magendie himself, if he had not taken the precaution of collecting the gases under mercury. Were we inclined to make another remark, it would be, how has Mr. M. arrived at the perfections to collect in analysis all the 100 parts? B.

---

*List of Patients admitted into all the Civil Hospitals of Paris, from the* 1st *of March, to the* 31st *of March,* 1817, *both inclusive.*

| | |
|---|---|
| Uncharacterised fevers | 26 |
| Intermittent ditto, various | 210 |
| Bilious or gastric ditto | 122 |
| Mucous ditto | 2 |
| Adynamic or putrid ditto | 18 |
| Ataxic ditto | 4 |
| Catarrhal ditto | 20 |
| Phlegmasiæ, internal and external | 125 |
| ——— of the organs of respiration | 126 |
| Phthisis | 24 |
| Diarrhœa and dysentery | 23 |
| Ophthalmia | 28 |
| Dropsy and anasarca | 38 |
| Apoplexy and recent paralysis | 21 |

| | |
|---|---|
| Variolous ........................................ | 8 |
| Sporadic and chronic disosders, and the result of accidents | 258 |
| Itch ........................................ | 100 |
| Rheumatism ........................................ | 14 |
| Erysipelas ........................................ | 7 |
| Metallic colics ........................................ | 2 |
| Total .. | 1177 |

*List of Patients admitted into all the Civil Hospitals of Paris, from the* 1st *of April* 1817 *to the* 30*th, inclusive.*

| *From April* 1, | 10 | 21 | 30 | |
|---|---|---|---|---|
| Uncharacterised fevers .................... | 8 | 4 | 10 | 22 |
| Intermittent do. of various types ........ | 61 | 38 | 72 | 171 |
| Bilious or gastric do. .................... | 37 | 41 | 54 | 132 |
| Mucous do. .................... | 0 | 3 | 0 | 3 |
| Adynamic or putrid do. .................... | 14 | 9 | 6 | 29 |
| Ataxic do. .................... | 1 | 0 | 0 | 1 |
| Catarrhal do. .................... | 2 | 2 | 0 | 4 |
| Phelgmasiæ, internal and external ........ | 45 | 81 | 145 | 269 |
| Ophthalmia .................... | 11 | 7 | 9 | 27 |
| Rheumatic pains .................... | 12 | 6 | 8 | 26 |
| Diarrhœa and dysentery .................... | 12 | 4 | 4 | 20 |
| Erysipelas .................... | 3 | 2 | 1 | 6 |
| Phlegmasia of the organs of respiration ... | 65 | 0 | 0 | 65 |
| Consumptions .................... | 18 | 11 | 8 | 37 |
| Apoplexy and recent paralysis ........ | 6 | 8 | 7 | 21 |
| Dropsy and anasarca .................... | 17 | 17 | 10 | 44 |
| Variolous .................... | 1 | 1 | 1 | 3 |
| Metallic colics .................... | 5 | 1 | 5 | 11 |
| Sporadic and chronic disorders and accidents | 75 | 72 | 77 | 224 |
| Itch .................... | 24 | 16 | 36 | 76 |
| | | | Grand Total ...... | 1192 |

*Meteorological Report.*

| *From April* 1, | 10 | 20 | 30 |
|---|---|---|---|
| Maximum of barometer ............ | 28.8l.$\frac{7}{12}$ | 28.7l. | 28.5$\frac{11}{12}$ |
| Minimum of do. .................... | 28.1l.$\frac{5}{12}$ | 27.11$\frac{6}{12}$ | 28 $\frac{1}{12}$ |
| Max. of thermometer .............. | 14°.8 | 11°. | 8°. 7 |
| Min. of do. .................... | 3°.3 | 0 $\frac{6}{12}$ | 4°. 9 |
| Max. hygrometer .................... | 89°.5 | 91°. | 92°. 5 |
| Min. of do. .................... | 86 .5 | 84° | 84° |

Chevallier, Ingenieur-opticien.

*Reigning Maladies in Paris from the* 1*st to the* 10*th of April.*

From the commencement of the month the weather has been constantly mild and serene, though the winds have been continually northerly or north-easterly; the centigrade thermometer was in the the morning at 5 to 6 degrees, and in the middle of the day 16, 17, and even 18°, or equal to about 12, 13, 14 of Reaumur.

This sudden change of temperature, and the passage directly from humid cold to dry heat, has produced many disorders; inflammations of every kind are become frequent, especially those of the organs of respiration, that is to say, catarrhs and violent colds, quinzies, pleurisies, and *pleuro-pneumonies.* Several persons have experienced sudden hæmorrhages either by the lungs or the uterus. The affluxes of the blood to the head, and the consequent accidents, apoplexies, paralysis, &c. have been pretty common; and it may be remarked, that the sudden appearance of fine weather has produced more disorders than existed during the intemperate cold and humidity with which we have been so long afflicted.

*From the 11th to the 20th of April.*

The change in the commencement of this decade has been as rapid as in the last; the weather has become cold and cloudy, the west and north winds very strong, and almost every day a fall of snow or hail,—the mercury has been even at the freezing point. This sudden change of temperature has produced a great quantity of pulmonary catarrhs, and the most serious disorders. The croup, to which the attention of parents ought perpetually to be awake, has carried off several victims; its symptoms are, a slight pain in the throat, a particular hoarseness of the voice, and convulsive cough, of which the fits are followed by a perfidious calm, during which the disorder makes a rapid progress, and is soon above all the resources of the art.

Another prevalent complaint is spontaneous and very violent hæmorrhage, principally from the uterus and the intestines; and, in younger subjects, from the nose.

*From the 21st to the 30th of April.*

The north winds have been very violent and the weather foggy, the winds cold and penetrating; but the number of catarrhs and acute affections of the breast are greatly diminished. The measles are very prevalent and intense, but regular. The most common disorders are colics and disorders of the bowels of all kinds, even to the inflammation of the peritoneum, intestinal hæmorrhages, costiveness, &c.

---

We have received a very long and dull article from Paris, on the antiquity of Cow-pox, and the knowledge of its prophylactic properties against the variolous poison. In our opinion, it were much more to the credit of antiquity, if all this was consigned to oblivion. The merit of a discovery is its application. We might as well say, Sir Isaac Newton's grandmother's gardener discovered the laws of gravitation, because he knew, before that philosopher was born, that apples fall to the ground and never rise spontaneously upwards. The following conclusion of the paper is creditable to the candour of the writers.

 "It

"It follows from this remarkable passage—1. That thirty-three years since the vaccine or variolous disorder of cows was known in this country, and principally in the environs of this place.—2. That the persons who milked them knew that it was contagious even for man.—3. That it was not considered a dangerous disorder.—4. That those who had it believed themselves thereby preserved from small-pox.—5. That this opinion was not confined to the credulous country-people only, but was common also to sensible persons residing in the country, and who considered it not as an opinion, but as a truth confirmed by long experience, and as a precious and important observation:—and 6 and lastly, That this important observation has been established long ago in a periodical publication.

In detailing these facts, we are far from seeking to diminish the glory of Jenner, who must ever be regarded as one of the greatest benefactors of humanity; but it is our duty to record historical facts connected with the science of medicine and the art of healing. Truth is an imperious obligation, and Dr. Jenner himself will always enjoy too much glory to covet one ray that does not belong to him.

---

### REPORT OF DISEASES.

Inflammatory diseases continue to rage; and, what would appear most extraordinary, even the poverty of the laboring classes proves no exemption. That this must partly arise from some constitution in the atmosphere can hardly be questioned, when we reflect, during a former period of distress, the sagacious Willan remarked, that, even in cases of apparently active delirium, to take only a very small quantity of blood by cupping was dangerous, but to bleed by the arm fatal. At present, to bleed is often the only means of preserving our patient. It is true, those of the above rank only require a very small abstraction, and the relief is very considerable.

Dysentery is sporadic, but for the most part mild, especially if early treated with evacuants. In the chronic stage, small doses of ipecacuanha retain their credit as a specific. Scarlatina and small-pox are still frequent; the former mild, but the latter in some cases very severe. Cases after vaccination still occur, but the good sense of the public, now that they are left to themselves, is sufficient to preserve the credit of that valuable discovery, which gains ground, though no sinister attempts are made at concealing the few instances in which it seems to fail.

The Reporter some time ago mentioned cases of frequent hæmorrhage from the uterus or vagina, attended with intense pain in the neighbouring parts, such as usually terminate in the malignant ulcer. These he had succeeded in treating with frequent cupping over the os coxigis, and leeches about the pubis and inguen. A

case of four years standing lately occurred, in which this mode of treatment has perfectly succeeded. Those who feel as they ought will not require to be informed of the gratitude of the patients for such relief, or their anxiety to return to the remedy on the slightest threat of pain.

Several cases of bronchitis, imitating phthisis, have occurred and yielded to purgatives united with diuretics. We have been informed, that the use of vinegar, as recommended in the last volume of Medical Transactions, has, in some cases of apparently the most serious form of phthisis, been found completely successful.

---

### Mr. Salisbury's *Botanical Excursions for May.*

May 8th.—Battersea fields, and the gardens in that neighbourhood.

Scandix odorata

Narcissus angustifolius

——— Pseudo Narcissus

——— biflorus

——— major

Milium effusum

Isatis tinctoria

Chelidonium majus

Chærophyllum temulentum

Sorbus Aucuparia

Mespilus Oxycantha

Sorbus hybrida

Galium palustre

——— erectum

Salix monandra

Alchemilla Alpina

——— arvensis

——— vulgaris

Valeriana locusta

——— rubra

Samolus valerandi

Ægopodium Podograria

Myosurus minimus

Poa angustifolia

Vicia bithinica

Orobus tuberosus

Vicia sativa

Polygonum Bistorta

Rumex Acetosella

Sambucus nigra

Prunus Padus

Pyrus Malus

Arum maculatum

Brassica Napus

Lycopsis arvensis

Draba muralis

May 16th.—Hampstead-heath and the neighbourhood.

Orchis maculata

——— mascula

——— morio

Stachys palustris

Poa loliacea

——— rigida

Medicago polymorpha

Lapsana communis

Erysimum Barbarea

Erysimum Alliaria

Poligonum aviculare

Alisma plantago

Potentilla alba

——— verna

——— aurea

——— rupestris

Convolvulus arvensis

Scilla nutans.

May 22d.—Charlton and neighbourhood.

Veronica spicata

Anemone Nemorosa

Sedum acre

——— rupestre

——— reflexum

——— album

Thlaspi campestre

Silene armeria

Ophrys bifolia

Adonis autumnalis

Cratægus torminalis

Iberis nudicaulis

——— amara

Salvia pratensis

Berberis vulgaris

Betula alba

Lolium perenne

Hypericum humifusum

Carex

Carex vaginata
—— curta
—— depauperata
Plantago lanceolata
Centunculus minimus
Scirpus fluitans
Potomageton natans
Carum Carui
Scandix Cerifolium
Parietaria officinalis
Statice Armeria
Festuca pratensis
——— elatior
Vaccinium myrtillus
Pedicularis palustris
Ajuga reptans
Lychnis viscaria
Acer Pseudo Platanus
Veronica serpyllifolia
Salix cineria
Hottonia palustris
Ilex aquifolium
Ulmus campestris
Genista anglica
Turritis glabra
Poa nemoralis
Fagus sylvatica
Carex panicea
Acer campestre
Medicago falcata
Melica uniflora
Mercurialis perennis.

N.B.—We have not collected all that are in bloom this month, and the next excursion we may expect will afford us a rich collection.

As my summer class is not yet completed, those gentlemen who wish to attend, may hear of the appointed days for June at the Botanic Garden, or by consulting the notices which I shall have posted at Guy's Hospital.

*May* 28th, 1817.

---

### CATALOGUE OF MEDICAL BOOKS.

### A METEOROLOGICAL JOURNAL,

By Messrs. William Harris and Co. 50, Holborn, London.

*From the 20th April to the 19th May, inclusive.*

| Day of Month. | Rain gauge | Therm. | | | Barom. | | De Luc's Hygrom. Dry | | Damp | | Wind. | | Atmospheric Variation. | | |
|---|---|---|---|---|---|---|---|---|---|---|---|---|---|---|---|
| April | | | | | | | | | | | | | | | |
| 20 | | 48 | 54 | 42 | $30^5$ | $30^5$ | 1 | 0 | | 0 | N va. | N va. | Fine | | Clo. |
| 21 | | 46 | 52 | 39 | $30^5$ | $30^4$ | 0 | 1 | 0 | | NE | E | Fine | | Clo. |
| 22 | | 44 | 51 | 39 | $30^4$ | $30^3$ | 1 | 1 | | | NE | ENE | Fine | | |
| 23 | | 41 | 50 | 34 | $30^3$ | $30^3$ | 1 | 0 | | 0 | NE | NE | Clo. | Rain | Fine |
| 24 | ,03 | 39 | 51 | 39 | $30^3$ | $30^3$ | 0 | 0 | 0 | 0 | NE | NNE | Clo. | Fine | Rain |
| 25 | ,02 | 43 | 49 | 39 | $30^3$ | $30^2$ | | | 1 | 1 | N va. | N | Clo. | | |
| 26 | | 44 | 56 | 39 | 30 | $29^9$ | 0 | 0 | 0 | 0 | N | NW | Clo. | | Rain |
| 27 | 05 | 49 | 59 | 39 | 30 | $30^1$ | 1 | 1 | | | N | SE | Fine | | |
| 28 | | 41 | 54 | 47 | $30^1$ | 30 | 1 | 1 | | | SW | NW | Fine | | |
| 29 | | 49 | 55 | 45 | $29^9$ | $29^8$ | 0 | | 0 | 1 | WSW | SW | Fog | Clo. | |
| 30 | ,05 | 47 | 54 | 41 | $29^7$ | $29^8$ | | | 1 | 1 | NNW | NW | Clo. | | Fine |
| May | | | | | | | | | | | | | | | |
| 1 | | 45 | 50 | 45 | $29^9$ | 30 | 0 | | 0 | 1 | N | NNE | Clo. | | Fine |
| 2 | | 47 | 53 | 42 | 30 | $29^9$ | | | 1 | 1 | N | NE | Clo. | Fine | |
| 3 | | 45 | 52 | 46 | $29^8$ | $29^8$ | | | 1 | 1 | NW | W | Clo. | Fine | |
| 4 | | 49 | 57 | 46 | $29^9$ | 30 | | | 1 | 1 | W | NW | Clo. | Fine | |
| 5 | | 59 | 65 | 47 | 30 | 30 | 1 | 1 | | | W | W | Fine | | |
| 6 | | 56 | 58 | 47 | 30 | $30^2$ | 1 | 1 | | | SW | E | Fine | | |
| 7 | | 49 | 57 | 48 | 30 | $29^8$ | 0 | 0 | 0 | 0 | NE | E | Clo. | Rain | Clo. |
| 8 | ,12 | 50 | 55 | 42 | $29^8$ | $29^8$ | 1 | | | 1 | E | NE | Clo. | | Rain |
| 9 | ,07 | 49 | 56 | 43 | $29^8$ | $29^7$ | | | 1 | 1 | NW | W | Clo. | | Rain |
| 10 | .09 | 45 | 59 | 46 | $29^5$ | $29^4$ | | | 1 | 1 | S | SW | Clo. | Rain | Clo. |
| 11 | ,05 | 47 | 57 | 47 | $29^2$ | $29^2$ | | | 1 | 1 | S | SW | Clo. | Fine | Clo. |
| 12 | | 49 | 56 | 47 | 29 | 29 | 0 | 0 | 0 | 0 | SSW | SW | Rain | Clo. | |
| 13 | ,13 | 53 | 57 | 50 | $29^2$ | $29^4$ | 0 | 0 | 0 | 0 | SSW | SW | Fine | | Clo. |
| 14 | ,06 | 57 | 58 | 50 | $29^5$ | $29^5$ | | | 1 | 1 | S | WSW | Clo. | | |
| 15 | | 54 | 60 | 52 | $29^4$ | $29^5$ | 1 | 1 | | | SW | W | Rain | Clo. | Rain |
| 16 | ,21 | 56 | 66 | 53 | $29^7$ | $29^9$ | 5 | 3 | | | SSW | S | Fine | | |
| 17 | | 49 | 66 | 50 | $29^6$ | $29^5$ | 1 | 1 | | | SW | SW | Fine | | |
| 18 | .16 | 54 | 64 | 45 | $29^4$ | $29^4$ | 0 | | 0 | 1 | W | NNW | Fine | Rain | Clo. |
| 19 | ,23 | 49 | 59 | 40 | $29^5$ | $29^5$ | | | 1 | 1 | N | NE | Clo. | Rain | |

The quantity of rain fallen in the month of April is only 24·100ths of an inch.

